# Supplementary material for: A Ruthenium(II) Polypyridyl Complex Disrupts Actin Cytoskeleton Assembly and Blocks Cytokinesis
Source: Angew Chem Int Ed Engl. 2022 May 3;61(27):e202117449. doi: 10.1002/anie.202117449 (PMC9323417; doi:10.1002/anie.202117449)

Angewandte Chemie
Eine Zeitschrift der Gesellschaft Deutscher Chemiker

Supporting Information

A Ruthenium(II) Polypyridyl Complex Disrupts Actin Cytoskeleton Assembly and Blocks Cytokinesis

M. R. Gill, P. J. Jarman, V. Hearnden, S. D Fairbanks, M. Bassetto, H. Maib, J. Palmer, K. R. Ayscough, J. A. Thomas*, C. Smythe**

Supporting Information for:

A Ruthenium(II) Polypyridyl Complex Disrupts Actin Cytoskeleton Assembly and Blocks Cytokinesis

Martin R. Gill,*[a] Paul J. Jarman,[b] Vanessa Hearnden,[c] Simon D Fairbanks,[d] Marcella Bassetto,[a] John Palmer,[b] Kathryn R. Ayscough,[b] Jim A. Thomas[d] and Carl Smythe*[b]

a Department of Chemistry, Faculty of Science and Engineering, Swansea University, Swansea, UK

b Department of Biomedical Science, University of Sheffield, Sheffield, UK

c Department of Materials Science and Engineering, University of Sheffield, Sheffield, UK

d Department of Chemistry, University of Sheffield, Sheffield, UK

* email c.g.w.smythe@sheffield.ac.uk or m.r.gill@swansea.ac.uk

Methods… 1

Supplementary Figures… 5

Supplementary Table… 14

Supplementary Video legends… 14

Supporting Information References… 14

Appendix 1: Original western blot data… 15

Appendix 2: NMR and MS data

Methods

Chemicals and antibodies

RuRuPhen and RuRuDMP were prepared and characterised as described previously.[1–3] Both complexes were used as racemic mixtures. RuRuPhen stock solutions of 10 mM in PBS were prepared and sterilized via filtration. Y-27632 (as the dihydrochloride) was purchased from Abcam. Nocodazole and Phalloidin-FITC were purchased from Sigma. Antibodies against cleaved caspase 3, p-MLC (Ser19), p-MLC (Thr18/Ser19), MLC, p-Chk1 (Ser345), pH2AX (Ser139), p-Chk2 (Ser516), Chk2 were obtained from Cell Signaling. Anti-β-actin and α-tubulin monoclonal antibodies were obtained from Sigma. p27Kip1 and Rho A antibodies were purchased from Santa Cruz.

Cell lines

A2780 and A2780-CP70 (CP70) cells were obtained from Professor Robert Brown, Imperial College London.[4] MCF7, A2780 and CP70 cells were cultured in RPMI medium supplemented with 10% FBS, 1 % (w/v) penicillin-streptomycin (Sigma) and 2 mM L-Glutamine (Sigma). HEK293 cells were cultured in DMEM medium supplemented with 10% FBS, 1 % (w/v) penicillin-streptomycin (Sigma) and 2 mM

L-Glutamine (Sigma). Cells were grown at 37 °C in a humidified 5 % CO_2 atmosphere and routinely sub-cultured using Trypsin.

Phalloidin and immunofluorescence staining

CP70 cells were seeded on Ibidi 35 mm μ-dishes (Thistle Scientific) and allowed to adhere for 24 h. After treatment as stated in the main text, cells were washed with PBS, fixed with 3.7% formaldehyde and membrane-permeabilized with 0.1% TRITON X-100 in PBS. Cells were washed in PBS before specific staining protocol.

Phalloidin staining: Samples were stained with 50 mg/ml Phalloidin-FITC in PBS (containing 1% DMSO from the original stock solution) for 40 minutes at room temperature in the dark. This solution was removed and the cells were washed with 3x PBS. Cells were stained with DAPI (5 ng/ml, 2 minutes), washed with PBS and then covered with PBS before visualization.

Immunofluorescence: Samples were blocked with 3% BSA (in PBS-T) for 1 h before incubation with anti-α-tubulin for 1 h (1/500 dilution in PBS-T). Samples were washed 3x in PBS-T and incubated with Alexa Flour 488-conjugated secondary antibody for 1 h (1/250 dilution). After further washing of 3x PBS, samples were co-stained with DAPI (5 ng/ml, 2 min), fresh PBS added and samples visualised by confocal microscopy. If not visualised on the day of staining, cells were stored at 4 °C in PBS and shielded from light.

Cell transfection

HeLa cells growing in 100 cm dishes were recovered by trypsinization, resuspended in 100 µl Neon Resuspension buffer (MPK10025T), and transferred in a Neon 100 µl pipette tip to a Neon electroporation chamber containing 3 ml of Neon Electroporation Buffer (MPK1096E). Electroporation was carried out using 2 pulses at 1005 V, 35mA. The tip containing cells was removed and the contents added to 5 ml of DMEM containing 5% fetal bovine serum and 6 µg of mRuby-LifeAct7 plasmid, before seeding at the required density. After 16 h, the cells were washed once in PBS prior to addition of fresh media.

Actin polymerisation analysis

In fluorometry assays, 5 µM purified rabbit skeletal muscle actin containing 5% pyrene-actin was incubated in 360 µl final volume of G buffer (2 mM Tris-HCL [pH 8.0], 0.2 mM $CaCl_2$, 1 mM NaN_3, 0.5 mM dithiothreitol, 0.2 mM ATP). Polymerisation salts were added to give the following final concentrations in a volume of 400 ml, 50 mM KCL, 1 mM $MgCl_2$, 1 mM EGTA, 10mM Tris-HCl [pH 8.0] before reading fluorescence. Polymerisation was observed in a Cary Eclipse fluorometer (emission 364 nm, slit 10 nm round; excitation 385 nm, slit 20 nm). High-speed centrifugation assays were performed as described previously.[5] Briefly, 5 µM actin was incubated alone in G buffer. 0.1 volumes of 10 x concentrated polymerization salts, (500 mM KCL, 10 mM $MgCl_2$, 10 mM EGTA, 100mM Tris-HCl [pH 8.0]), were added, followed by addition of RuRuPhen at indicated concentrations and times. Samples were then removed to ice and subjected to ultracentrifugation at 350,000 x g for 15 min at 4 °C in a Beckman Optima TL-100 ultracentrifuge using a TLA-100 rotor. The supernatant was carefully

removed, the pellet resuspended in an equivalent volume of G buffer, and protein content determined by Bradford assay, prior to electrophoresis on a 10% polyacrylamide gel.

ICP-MS analysis of cellular metal content

This was performed essentially as described in Jarman *et al*.[6] Briefly, A2780-CP70 cells were grown on 6-well plates at a seeding density of 0.75 x10^4 cells per dish and incubated for ~24 h until 50 -80% confluent. Cells were then treated with 100 μM RuRuPhen and incubated for the indicated times. At the indicated times, dishes were transferred to ice and medium was removed, cells were washed three times with ice-cold PBS and 1 mL of ice-cold serum-free medium and trypsin solution added. Dishes were incubated for 3 min and shaken to remove cells (plus scraped to detach any remaining cells) which were transferred to microcentrifuge tubes and centrifuged (4000 rpm, 3 min). The supernatant was removed, pellet resuspended in 500 µL serum-free medium and cells counted using a haemocytometer. Each sample was transferred to a glass sample tube, 2 mL concentrated HNO_3 added, heated to 60°C overnight and then diluted to 10 mL total volume with ultrapure Milli-Q H_2O before analysis of ruthenium content by inductively coupled plasma mass spectrometry (ICP-MS). Using the obtained ruthenium concentration, the sample volume, number of cells per sample and the assumption of a cell volume of 2 x10^{-12} L an estimate of intracellular concentration (mol L^{-1}) of RuRuPhen, which contains two ruthenium atoms per molecule, could be deduced.

Molecular Modelling

Molecular modelling experiments were performed on Asus WS X299 PRO Intel® i9-10980XE CPU @ 3.00GHz x 36 running Ubuntu 18.04 (graphic card: GeForce RTX 2080 Ti). Molecular Operating Environment (MOE, 2019.10, Montreal, QC, Canada)[7] and Maestro (Schrödinger Release 2020-2, New York, NY, USA)[8] were used as molecular modelling software. The chemical structure of RuRuphen was built and minimised in MOE 2019.10, then saved in sdf format for the docking analyses using the Maestro platform. Angle bonds and lengths for RuRuphen were checked against the crystal structure of a related Ru(II) complex with the same d^2sp^3 geometry and analogous ligands.[9] Actin crystal structures 1IJJ, 2Q0U, 2ASM and 2A42 were downloaded from the PDB (https://www.rcsb.org/) and prepared using the Protein Preparation tools in MOE 2019.10. The co-crystallised ligands were used to define the centre for the docking grid in Maestro. The protein–ligand complexes were saved in mae format, and pre-processed in Maestro using the Schrödinger Protein Preparation Wizard tool, by assigning bond orders, adding hydrogens and performing a restrained energy minimisation of the added hydrogens using the OPLS_2005 force field. 36 Å docking grids (inner-box 20 Å and outer-box 56 Å) were prepared using as the centroid the respective ligand. RuRuPhen was docked on the inhibitor sites using the Glide SP (standard precision) docking algorithm in the rigid docking mode,[8] keeping the default parameters, setting to 5 the number of output poses to include in the solution. No poses could be obtained for each of the three sites analysed with Glide SP. A blind docking study was conducted for RuRuPhen on the 2A42 crystal structure in MOE 2019.10, keeping the default parameters, setting the refinement of docking poses to none, and setting to 30 the number of output poses to include. The docking results were visually inspected in MOE 2019.10, prioritising the poses with the best (lowest) values of docking score.

Migration assay

Cell migration was measured using the Oris™ cell migration assay (Platypus Technologies, Madison USA). CP70 cells were seeded into 96-well plates and a circular exclusion zone was created using a stopper to prevent cell adherence in the centre of the well as per the manufacturer's guidelines. Once adhered, cells were treated with 0.5 µg/mL mitomycin C (Sigma, Dorset, UK) for 4 h to inhibit cell division, and the stopper was removed to create an exclusion zone of 5.37 ± 0.05 mm^2 that was imaged using a Spot™ USB camera (Spot Imaging Solutions, Michigan, USA) at baseline and following cell migration after 48 h. Area infiltrated was calculated as exclusion zone area (48 h incubation)/exclusion zone area (0 h incubation).

Cell proliferation and cell viability

CP70 cells were seeded in 6 well plates before treatment with solutions of RuRuPhen for 24 or 48 h. Cells were detached by scraping and concentrated via centrifugation. Pellets were re-suspended in 1 ml of serum-free medium, a sample of which was stained with Trypan Blue solution (0.4 %) and the number of total cells and Trypan Blue-positive cells counted by haemocytometer. A minimum of 200 cells were counted. For each independent experiment, counts were made in triplicate and an average value used. MTT assays were performed as described in a recent publication.[10] IC_{50} values were obtained by extrapolation of the concentration at 50% cell viability.

Flow cytometry

Cells were treated with 100 µM RuRuPhen for the time stated. Samples were harvested by trypsinization, washed with PBS and fixed with 70 % cold ethanol. Cells were RNAase treated, stained with 30 µg /mL propidium iodide (1 h at room temperature) and the DNA content analysed using a Biosciences LSRII Flow Cytometer. Data were processed for cell cycle phase apportionment using FloJo software.

Western blotting

Cells were grown and treated in 6 well plates. Cells were washed with cold PBS, detached using a cell scraper and lysed in lysis buffer (20 mM Tris, pH 7.5, 0.27 M sucrose, 1 mM EDTA, 1 mM EGTA, 1% Triton X-100, protease inhibitors (10 µg/mL leupeptin, 2 µg/mL pepstatin, 50 µg/mL antipain, 2 µg/mL aprotinin, 20 µg/mL chymostatin and 2 µg/mL benzamidine), and phosphatase inhibitors (50 mM NaF, 1 mM Na3VO4 and 20 mM Na β-glycerophosphate)) via freeze-thawing cycles (x3). Protein content of lysates was quantified via Bradford assay. Aliquots of cell lysates were resolved by 8-15% SDS-PAGE and transferred onto nitrocellulose membrane. Membranes were blocked with either 5% nonfat milk or 5% BSA (in TBST) before being probed with the appropriate antibodies dissolved in either 5% nonfat milk or 5% BSA (in TBST). Cell Signaling antibodies were used at the recommended dilution (typically 1/1000). Anti-β-actin and α-tubulin monoclonal antibodies were used at 1/5000 dilution. HRP-conjugated anti–rabbit or anti–mouse secondary antibodies (Santa Cruz Biotech.) were used at 1/5000

dilution. Blots were visualized using ECL or ECL+ chemiluminescence reagents (GE Healthcare Life Sciences) with X-ray development (Fuji medical film and Optimax 2010 processor).

Microscopy

Fluorescence microscopy was conducted on a Zeiss LSM 510 inverted confocal fluorescence microscope equipped with an Ar (458, 477, 488, 514 nm) 30 mW laser and 40x/1.3 oil immersion and 63x/1.4 oil immersion lenses. DAPI was excited using a 405 nm diode laser and emission detected using a 420-480 nm (blue) BP (band pass) filter. FITC-conjugated secondary antibodies were visualized using 488 nm Ar-ion excitation and a 505-550 nm (green) BP filter. Image data acquisition and processing was performed using Zeiss LSM Image software or ImageJ. Live cell (time lapse) imaging was performed on a Leica AF6000LX inverted microscope equipped with a 40x oil-immersion objective and an environmental chamber maintained at 37°C and 5% CO_2. Transmitted Light BrightField images were obtained. No adverse responses typical for RPC phototoxicity (cell death, oncosis etc.) were observed. Transmission electron microscopy on CP70 cells treated with RuRuPhen was performed as described in a previous publication, including sample preparation.[11] No other stain (e.g. uranyl acetate, osmium tetroxide etc.) was employed. Following processing, cells were viewed at 100 kV in a Hitachi H7600 transmission electron microscope.

Supplementary Figures

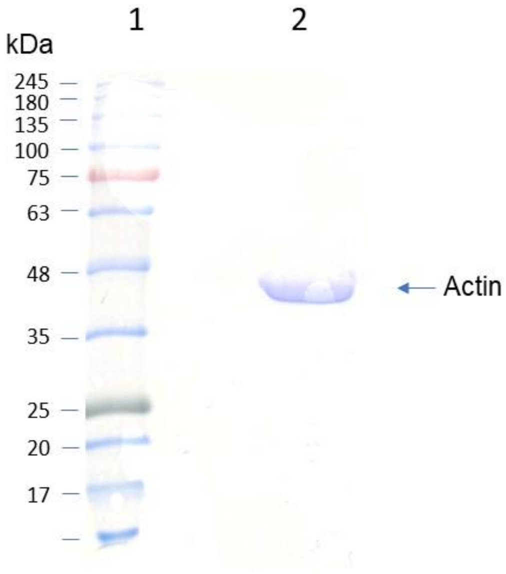

Figure S1. SDS-PAGE of purified rabbit skeletal muscle actin used in this study. Actin (5 μg) was subjected to electrophoresis in a 10% polyacrylamide gel, before staining with Coomassie Brilliant blue. Lane 1: Molecular weight markers. Lane 2: actin.

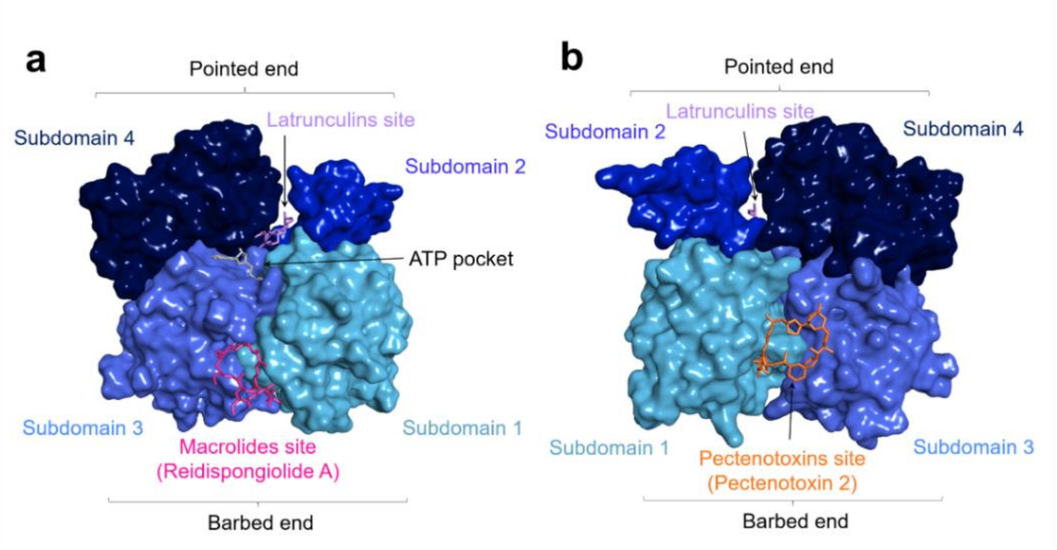

Figure S2. a) Surface representation of actin subdomains and binding pockets highlighting the ATP (atoms in grey) and latrunculins (atoms in lilac, latrunculin A) (PDB ID 1IJJ), and the macrolides binding sites (atoms in pink, reidispongiolide A, ligand coordinates from PDB ID 2ASM). b) Surface representation of actin subdomains and binding pockets (PDB ID 1IJJ), highlighting the pectenotoxins binding site (atoms in orange, pectenotoxin 2, ligand coordinates from PDB ID 2Q0U).

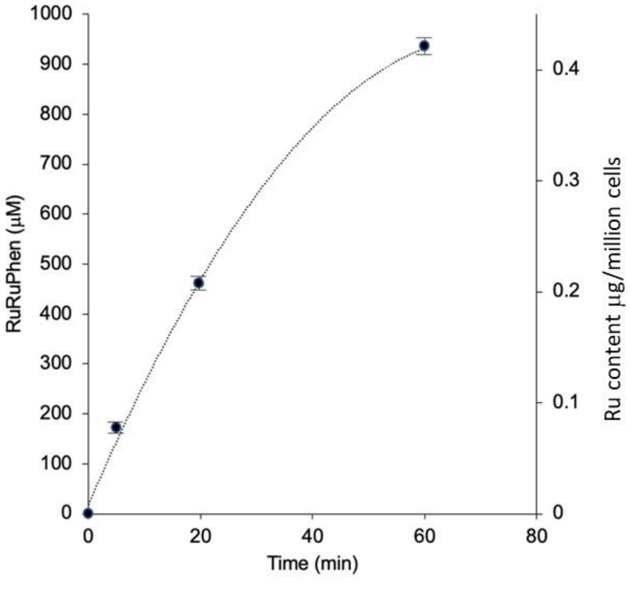

Figure S3. Cellular uptake of RuRuPhen in treated CP70 cells, as determined by ICP-MS analysis (100 μM for the indicated incubation times). Left hand axis, estimated intracellular concentration of RuRuPhen. Right hand axis, ruthenium content as μg Ru/million cells.

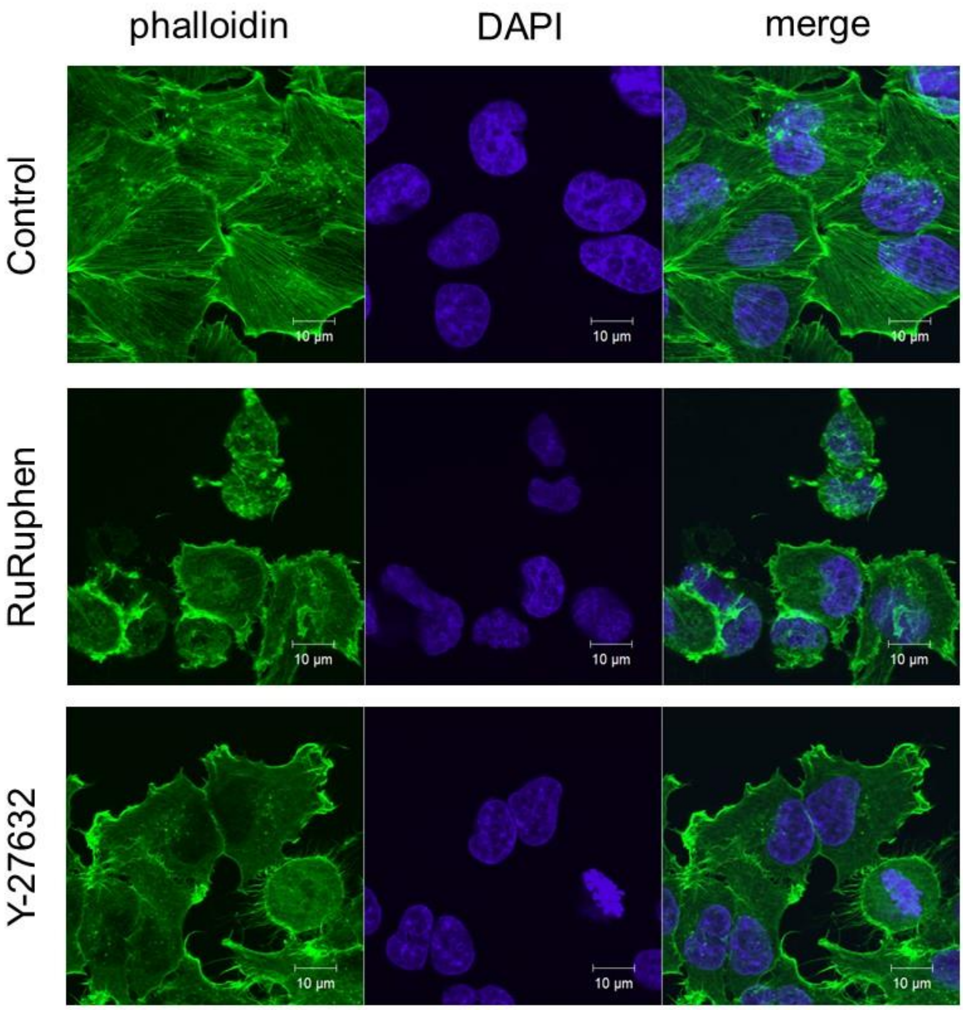

Figure S4. Confocal images of the actin cytoskeleton in RuRuPhen-treated CP70 cells (100 μM, 1 h), as determined by phalloidin staining. Y-27632 was employed as a positive control for actin stress fibre disruption.

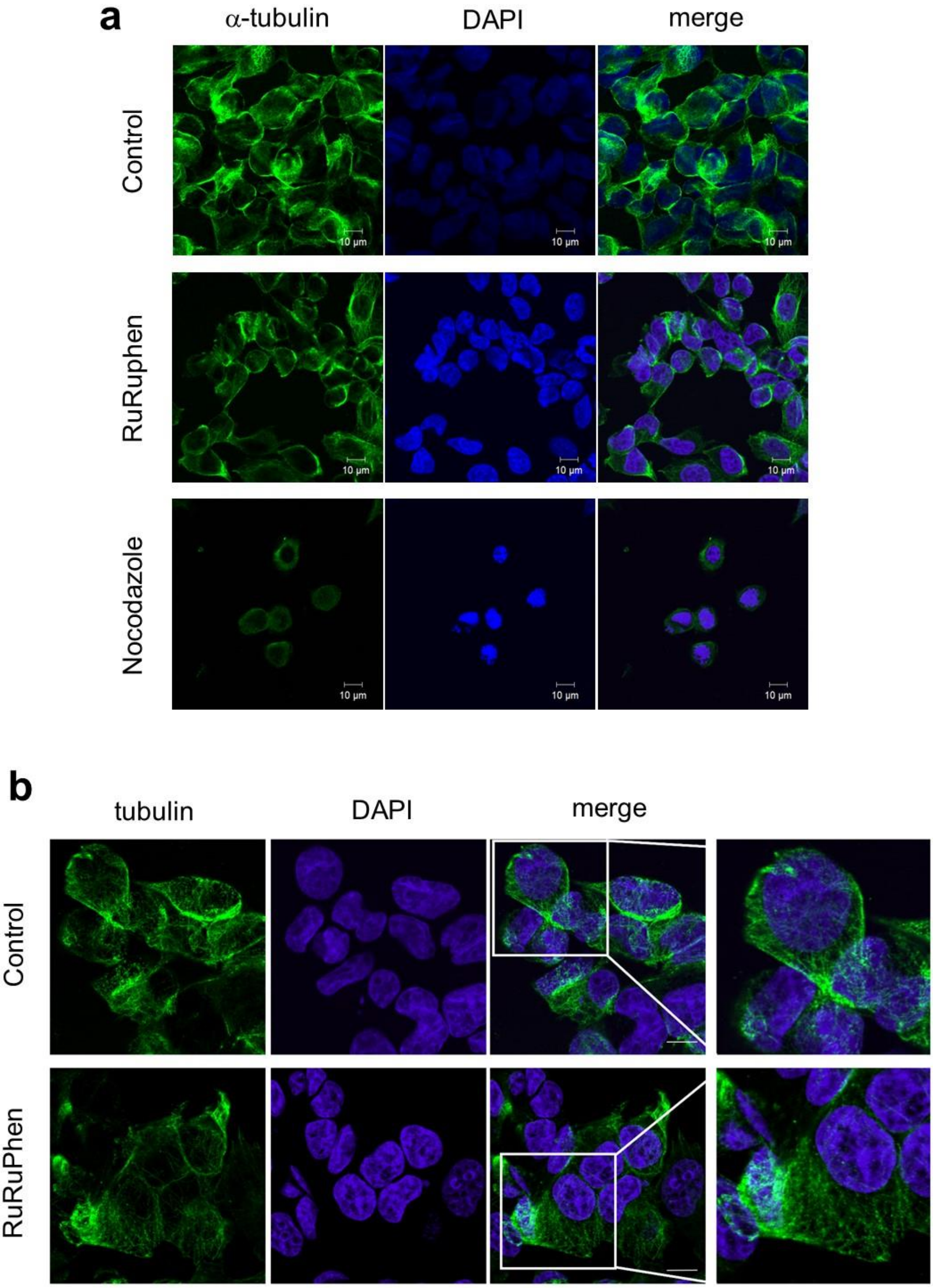

Figure S5. Confocal images of the tubulin cytoskeleton in RuRuPhen-treated CP70 cells (100 μM, 1 h), as determined by immunofluorescence. Nocodazole (500 nM, 1 h) was employed as a positive control for tubulin disruption. Scale bars in b = 10 μm.

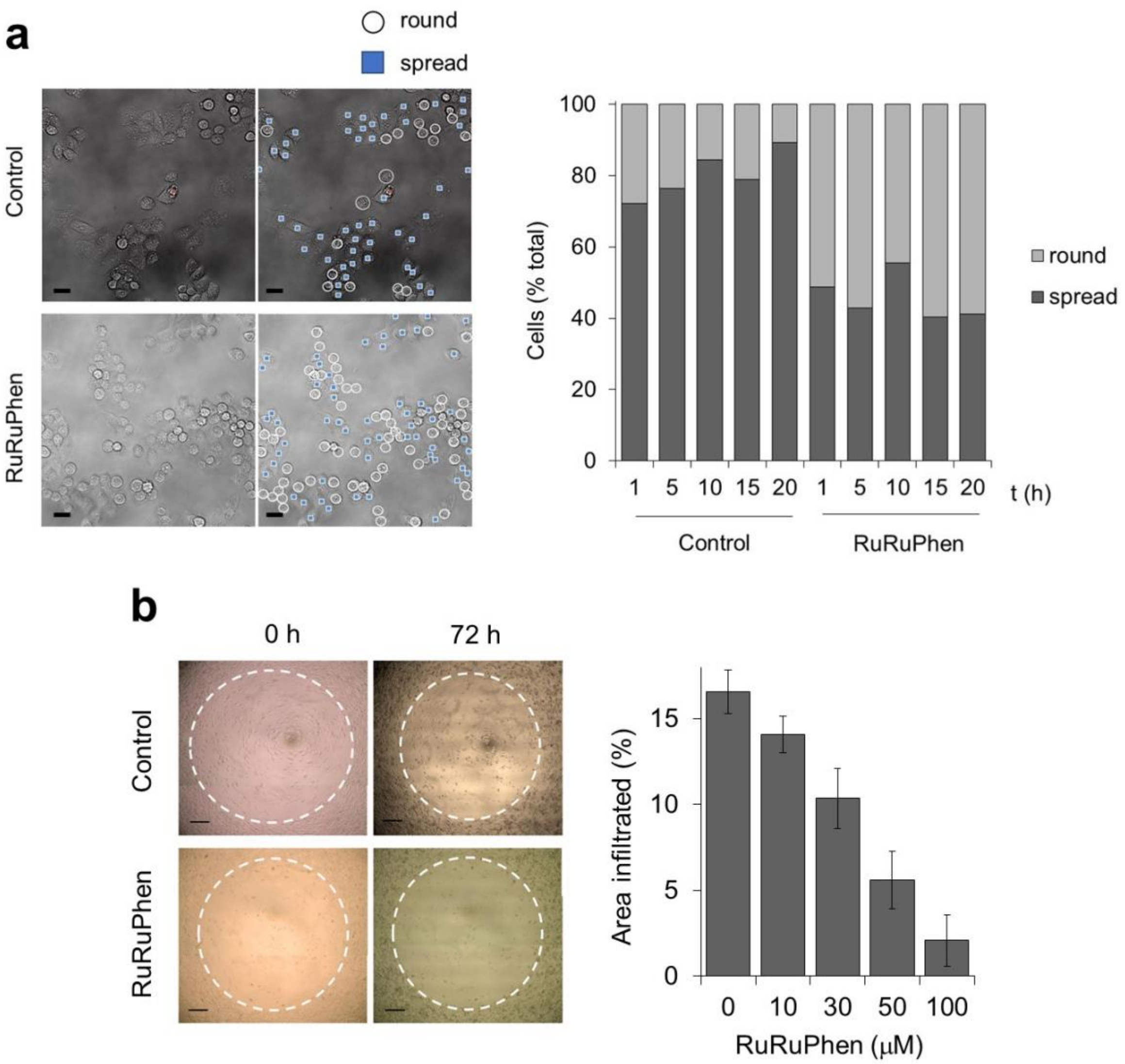

Figure S6. a) Temporal impact of RuRuPhen (100 μM) on CP70 cell morphology. Cells were scored as either a "spread" or "round" morphology. Scale bars = 20 μm. b) Impact of RuRuPhen on cell motility, as determined by migration assay. Area of migration quantified from three independent experiments and expressed as % of area infiltrated. Scale bars = 200 μm.

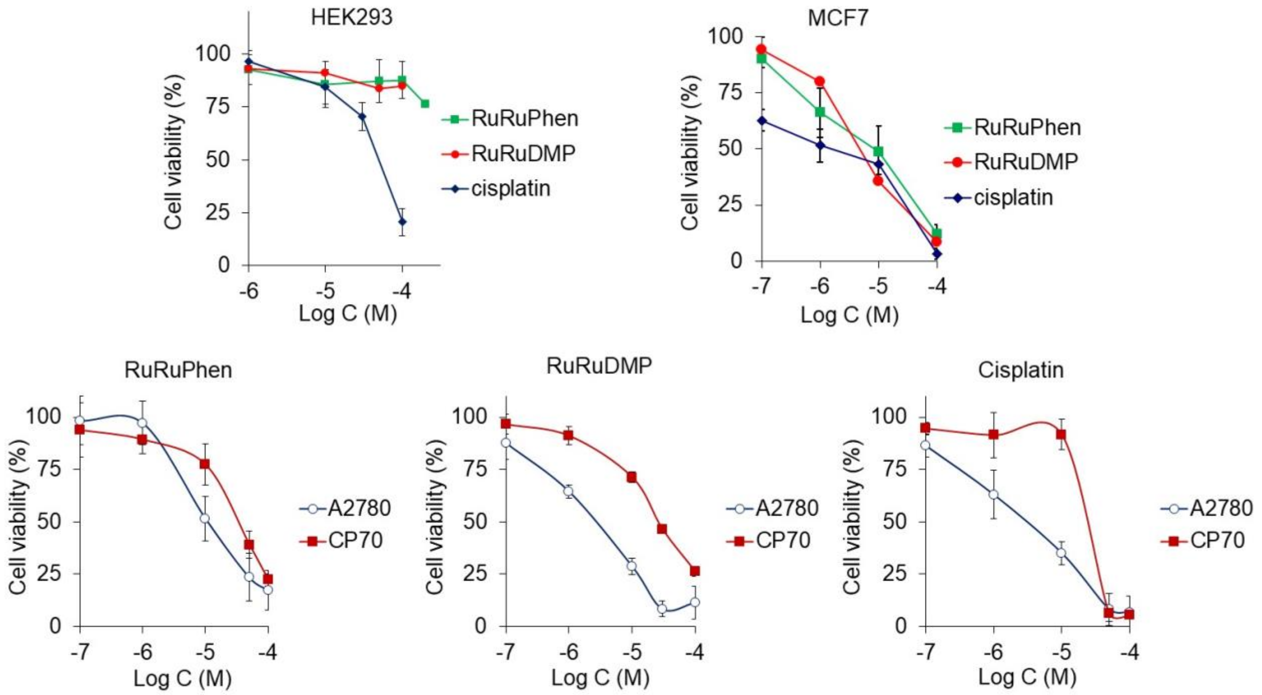

Figure S7. Top row: Impact of RuRuPhen, RuRuDMP or cisplatin on cell viability of HEK293 (48 h incubation, left) or MCF7 (72 h incubation, right) cells. Bottom row: Impact of RuRuPhen, RuRuDMP or cisplatin on A2780 or A2780-CIS cells (48 h incubation). % viability determined by MTT assay and normalised to a negative control for each experiment. Data is mean +/- S.D. of two or three independent experiments, where each condition was performed in triplicate.

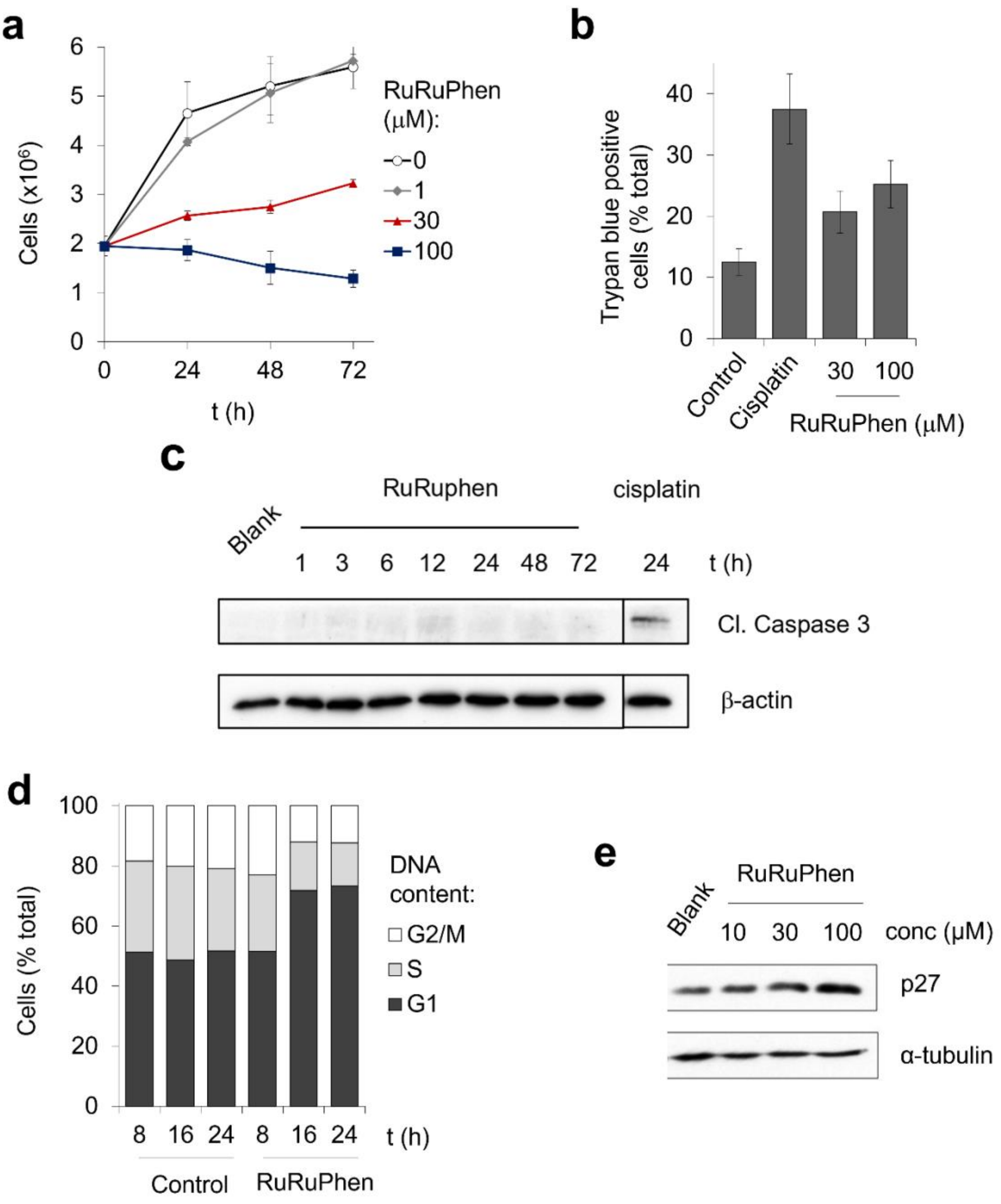

Figure S8. a) Impact of RuRuPhen on CP70 cell proliferation. Viable cells counted by Trypan Blue exclusion assay. b) Trypan Blue positive (i.e. non-viable) CP70 cells after treatment with cisplatin (40 μM) or RuRuPhen for 48 h. Data expressed as percentage of total cells counted, independent of Trypan Blue staining. Average of triplicates. c) Levels of apoptosis marker cleaved caspase-3 in cells treated with cisplatin (40 μM) or RuRuPhen (100 μM) for the time indicated, as determined by immunoblotting. β-actin levels were used as a loading control. The results for cisplatin and RuRuPhen were obtained from the same membrane, processed in an identical manner, and the splice is indicated by a border. d) Cell-cycle distribution of cells treated with RuRuPhen for 8, 16 or 24 h, as determined by DNA content and flow cytometry. e) p27 levels in RuRuPhen-treated cells, as determined by immunoblotting.

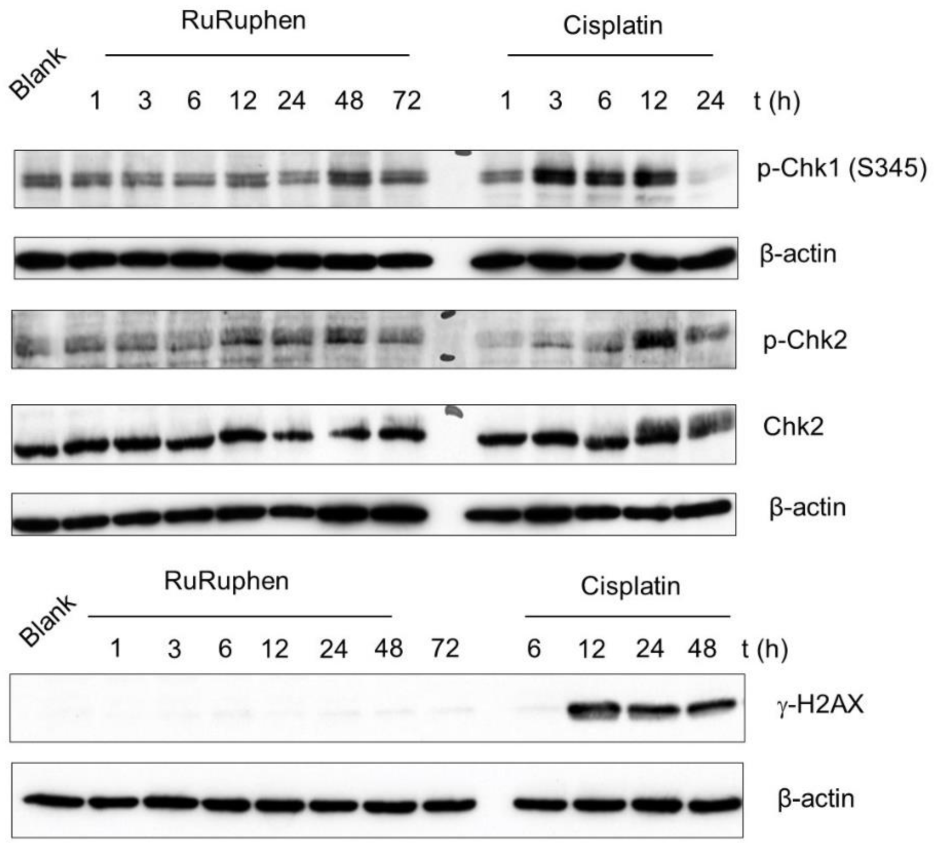

Figure S9. Whole-cell extracts of CP70 cells treated with RuRuPhen (100 μM) or cisplatin (40 μM) for the time stated were immunoblotted for activated (phosphorylated, p) p-Chk1 (Ser345), p-Chk2 (Thr68), or γH2AX (pH2AX at Ser139), as indicated. Total Chk2 protein levels independent of phosphorylation status are shown. β-actin levels were used as loading controls.

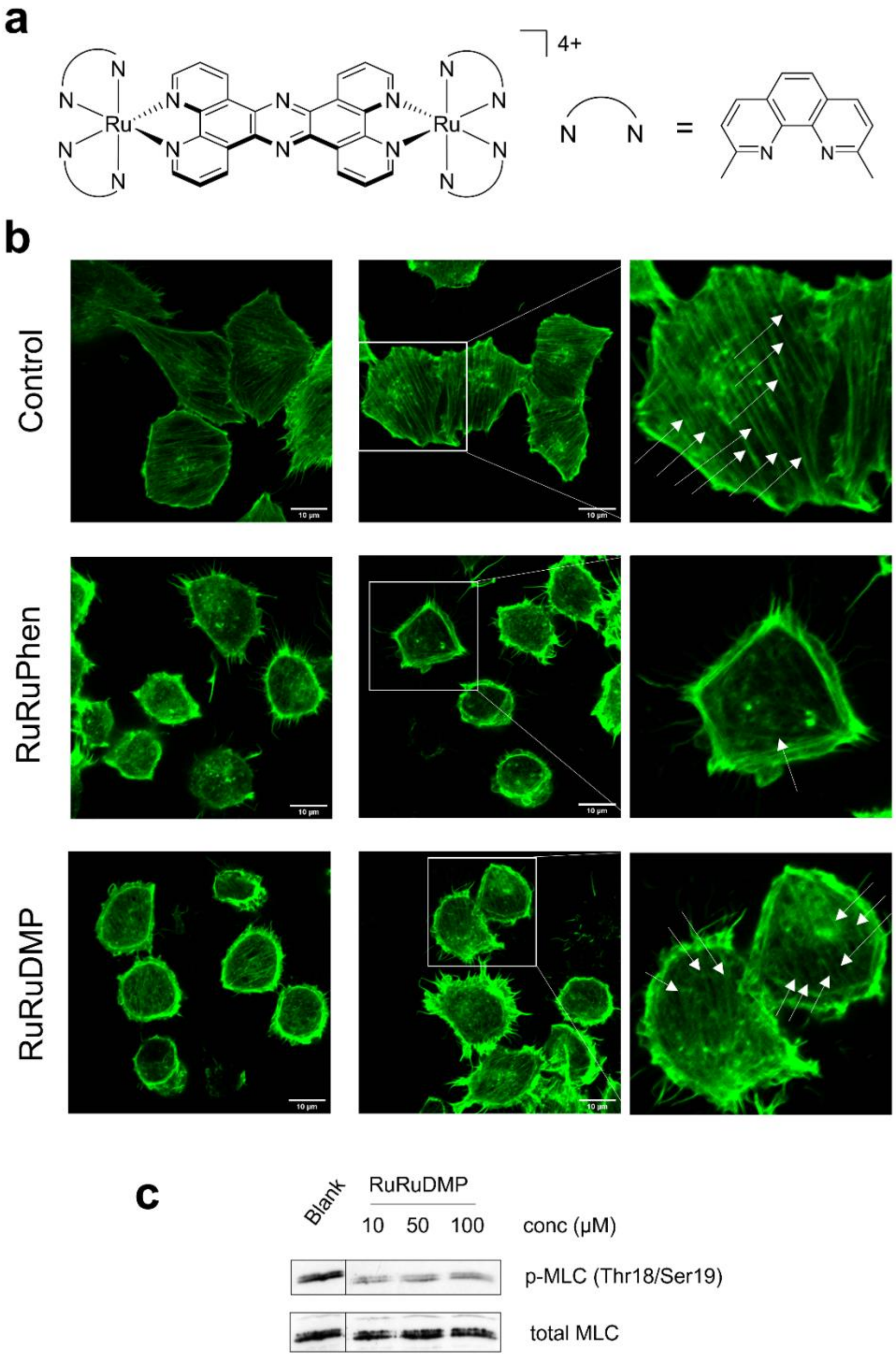

Figure S10. a) Structure of RuRuDMP. b) Impact of RuRuPhen or RuRuDMP (100 μM, 1 h) on the actin cytoskeleton of CP70 cells, as determined by phalloidin staining and confocal microscopy. Right hand side: High-magnification image showing actin stress fibres (arrows). Scale bars = 10 μm. c) p-MLC2 (MLC2 phosphorylated at Thr18/Ser19) levels in cell lysates of cells treated with indicated concentrations of RuRuDMP (24 h). Levels of MLC independent of phosphorylation status are shown. The results for blank (untreated cells) and RuRuDMP-treated were obtained from the same membrane, processed in an identical manner, and the splice is indicated by a border.

Supplementary Table

Table S1. Half inhibitory (IC_{50}) concentrations of RuRuPhen or RuRuDMP towards HEK293 cells (48 h incubation), MCF7 (72 h incubation), A2780 and CP70 cells (48 h incubation). Cisplatin treatment included for comparison. Data mean +/- S.D. of two or three independent experiments, where each condition was performed in triplicate.

Complex	HEK293	MCF7	A2780	A2780-CP70
RuRuPhen	>200	6 ± 2	12 ± 3	37 ± 5
RuRuDMP	>200	5 ± 2	3 ± 1	28 ± 1
Cisplatin	50 ± 5	6 ± 2	4.3 ± 0.8	25 ± 1

Supplementary Video legends

Video S1. Time-lapse movie of untreated CP70 cells, 0 – 20 h.

Video S2. Time-lapse movie of untreated CP70 cells, 0 – 20 h.

Video S3. Time-lapse movie of CP70 cells treated with 100 µM RuRuPhen, 0 – 20 h.

Video S4. Time-lapse movie of CP70 cells treated with 100 µM RuRuPhen, 0 – 20 h.

Supporting Information References

[1] C. Rajput, R. Rutkaite, L. Swanson, I. Haq, J. A. Thomas, *Chem. - A Eur. J.* **2006**, *12*, 4611–4619.

[2] J. Bolger, A. Gourdon, E. Ishow, J.-P. Launay, *Inorg. Chem.* **1996**, *35*, 2937–2944.

[3] K. L. Smitten, H. M. Southam, J. B. de la Serna, M. R. Gill, P. J. Jarman, C. G. W. Smythe, R. K. Poole, J. A. Thomas, *ACS Nano* **2019**, *13*, 5133–5146.

[4] S. T. Durant, M. M. Morris, M. Illand, H. J. McKay, C. McCormick, G. L. Hirst, R. H. Borts, R. Brown, *Curr. Biol.* **1999**, *9*, 51–54.

[5] S. J. Winder, L. Hemmings, S. K. Maciver, S. J. Bolton, J. M. Tinsley, K. E. Davies, D. R. Critchley, J. Kendrick-Jones, *J. Cell Sci.* **1995**, *108*, 63–71.

[6] P. J. Jarman, F. Noakes, S. Fairbanks, K. Smitten, I. K. Griffiths, H. K. Saeed, J. A. Thomas, C. Smythe, *J. Am. Chem. Soc.* **2019**, *141*, 2925–2937.

[7] Molecular Operating Environment (MOE 2019.10); Chemical Computing Group, Inc.: Montreal, QC, Canada. Available online: http://www.chemcomp.com (accessed on 16 January 2021)

[8] Schrödinger Release 2020-2; Glide Schrödinger LLC: New York, NY, USA, 2020. Available online: https: //www.schrodinger.com/maestro (accessed on 16 January 2021).

[9] T. Takase, R. Abe, D. Oyama, *Acta Crystallogr. Sect. E Crystallogr. Commun.* **2018**, *74*, 1097–1100.

[10] V. Ramu, M. R. Gill, P. J. Jarman, D. Turton, J. A. Thomas, A. Das, C. Smythe, *Chem. – A Eur. J.* **2015**, *21*, 9185–9197.

[11] M. R. Gill, J. Garcia-Lara, S. J. Foster, C. Smythe, G. Battaglia, J. A. Thomas, *Nat. Chem.* **2009**, *1*, 662–667.

Appendix 1: Original western blot data.

Bl = blank (negative control, untreated cells), L = ladder. All scans were converted to greyscale. Crop areas are approximate, and images were cropped after auto contrast/brightness functions performed.

Figure 4c, left:

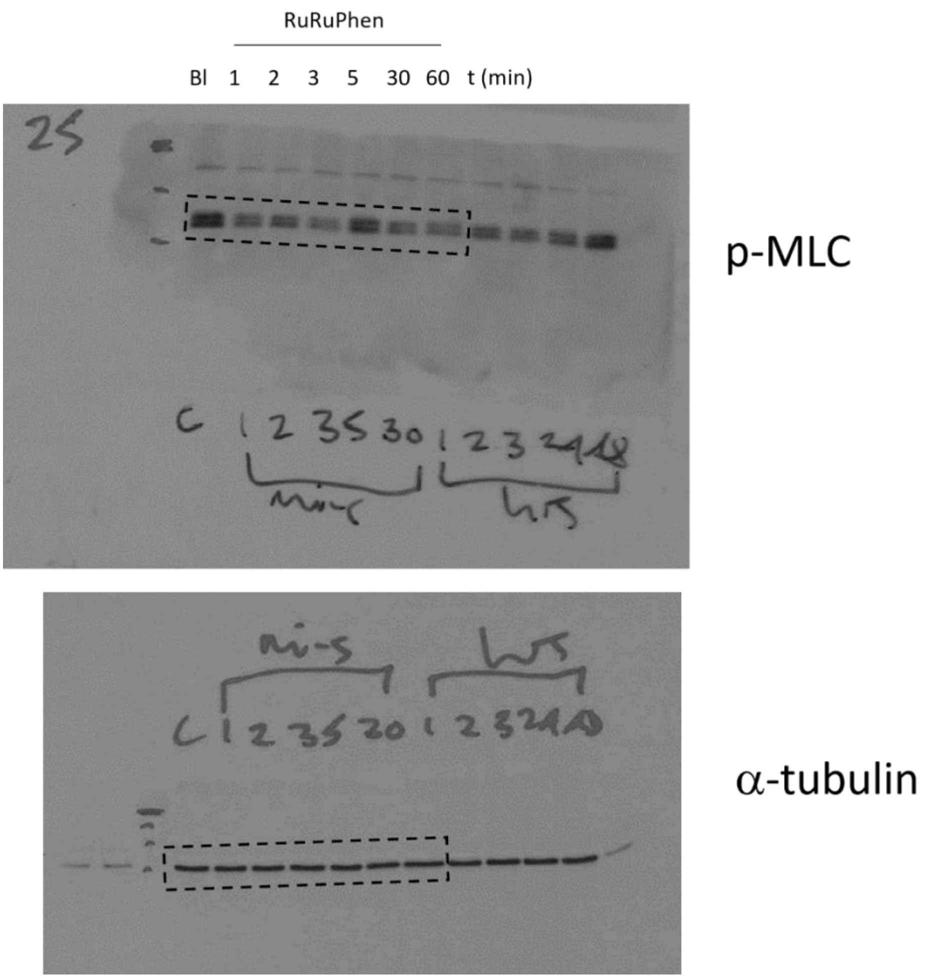

Figure 4c, right and Figure S10c:

RuRuPhen RuRuDMP

Bl Bl 10 50 100 10 50 100 (μM)

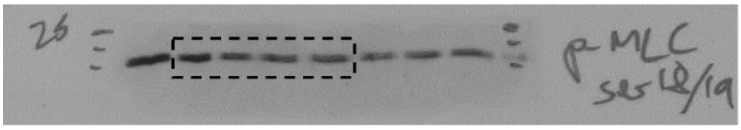

p-MLC

RuRuPhen RuRuDMP

Bl Bl 10 50 100 10 50 100 (μM)

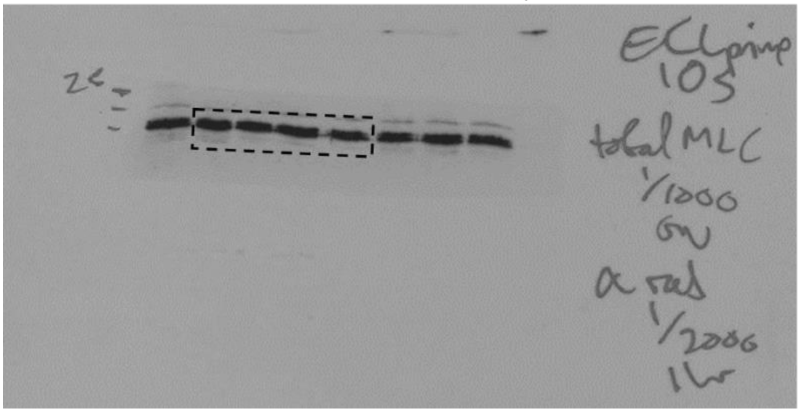

MLC

RuRuPhen RuRuDMP

Bl Bl 10 50 100 10 50 100 (μM)

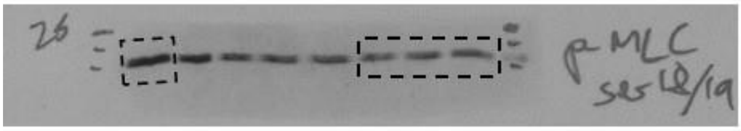

p-MLC

RuRuPhen RuRuDMP

Bl Bl 10 50 100 10 50 100 (μM)

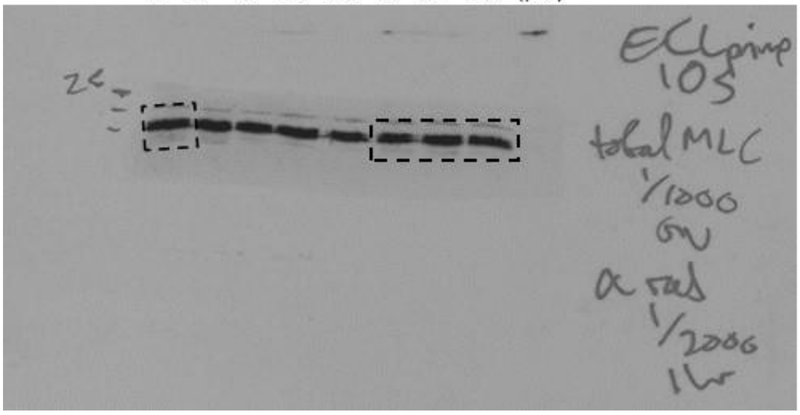

MLC

Figure S8c:

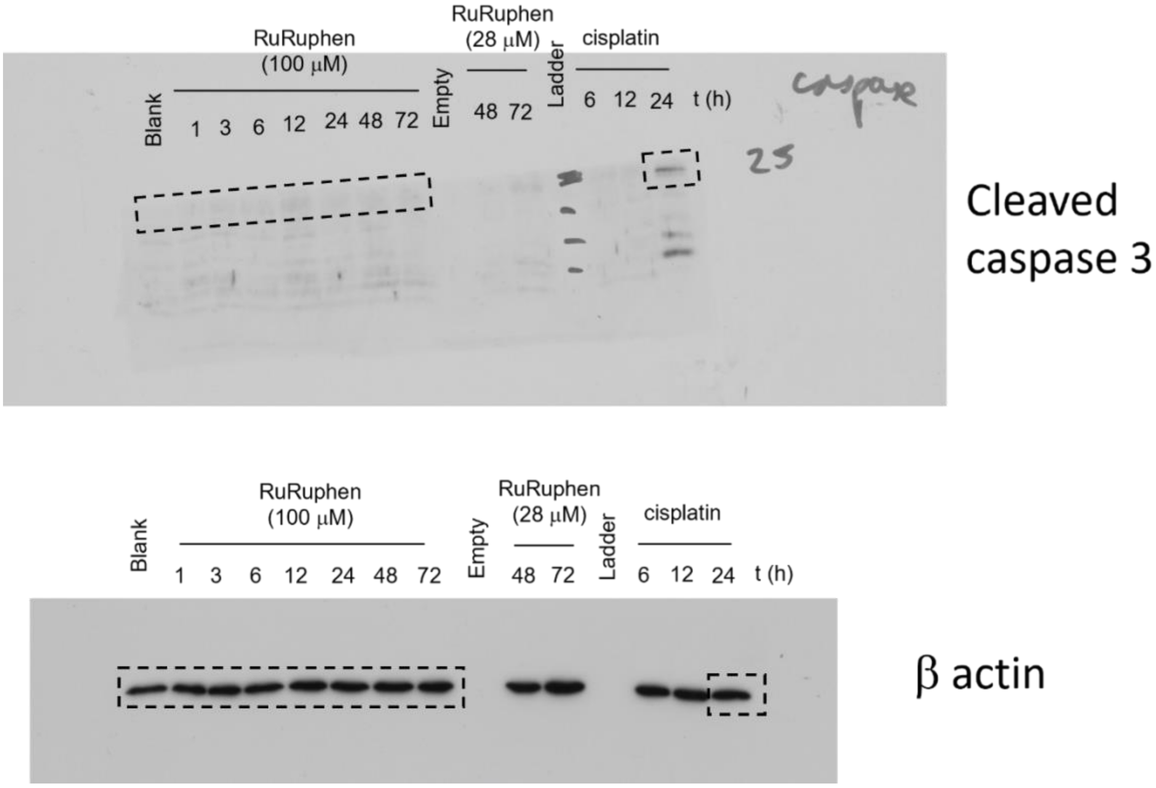

Figure S8e:

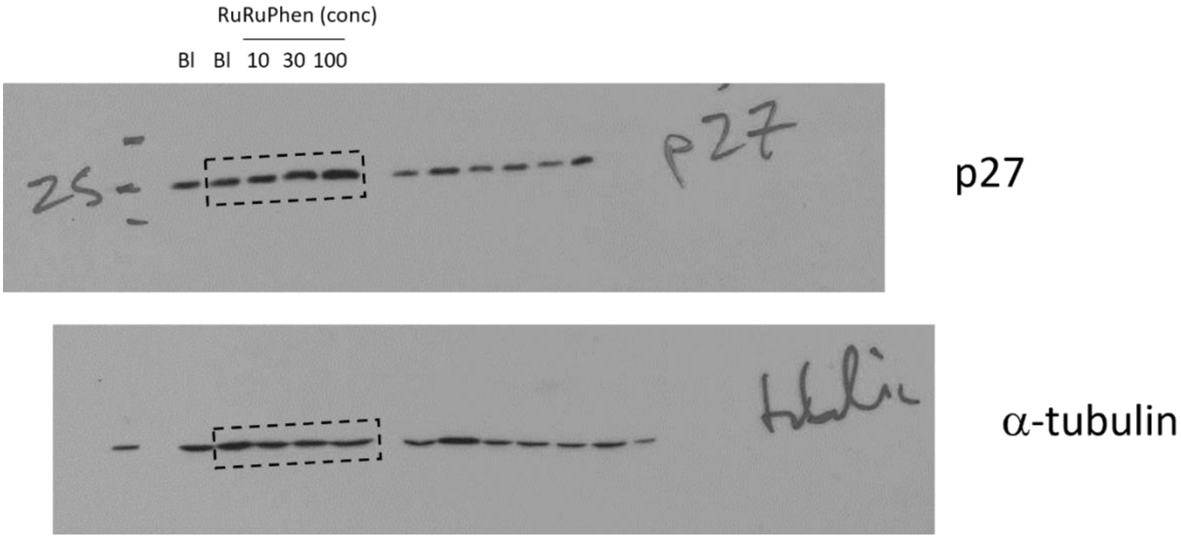

Figure S9:

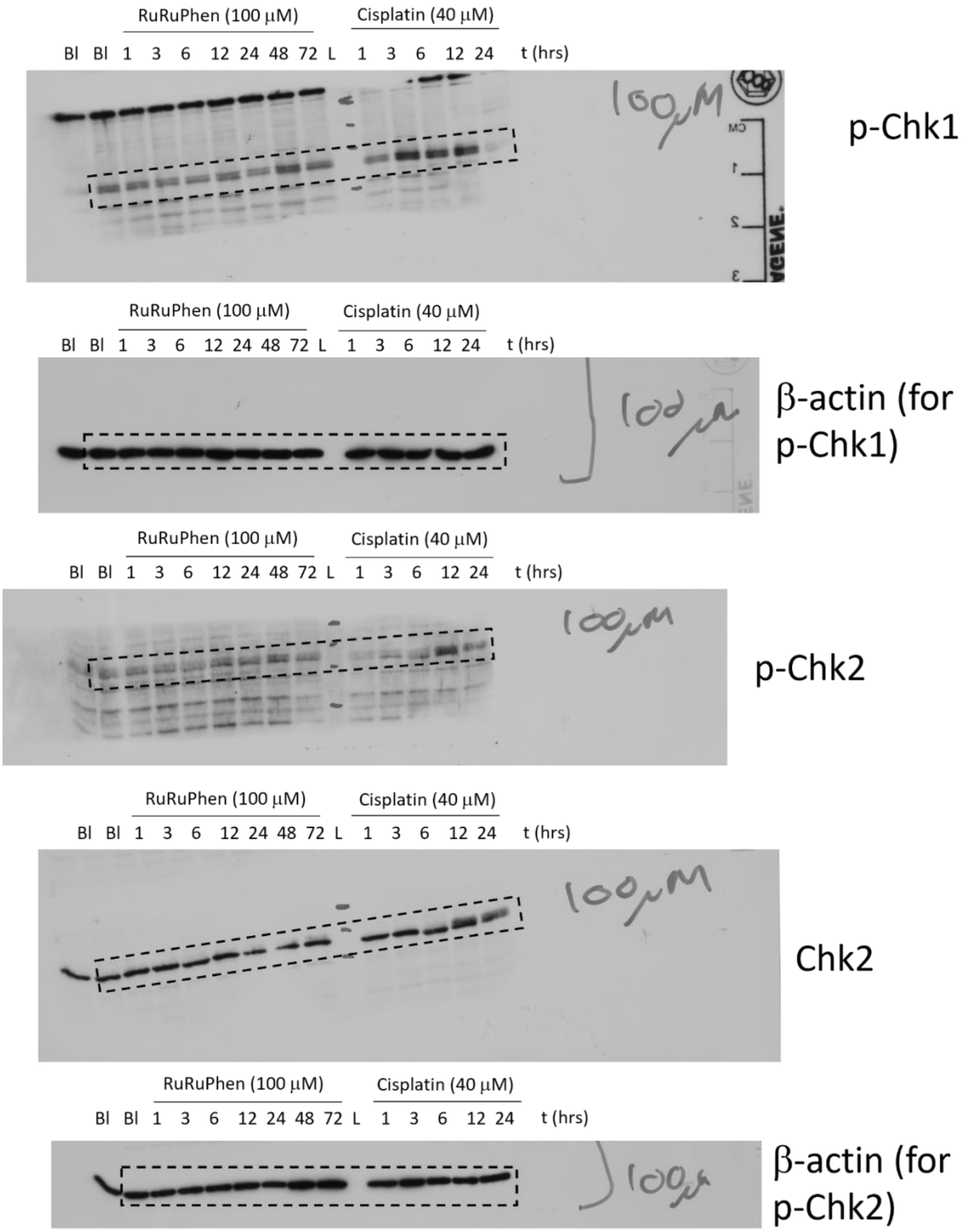
RuRuPhen (100 μM)
Cisplatin (40 μM)
Bl Bl 1 3 6 12 24 48 72 L 1 3 6 12 24 t (hrs)
100 μM
p-Chk1
RuRuPhen (100 μM)
Cisplatin (40 μM)
Bl Bl 1 3 6 12 24 48 72 L 1 3 6 12 24 t (hrs)
100 μM
β-actin (for p-Chk1)
RuRuPhen (100 μM)
Cisplatin (40 μM)
Bl Bl 1 3 6 12 24 48 72 L 1 3 6 12 24 t (hrs)
100 μM
p-Chk2
RuRuPhen (100 μM)
Cisplatin (40 μM)
Bl Bl 1 3 6 12 24 48 72 L 1 3 6 12 24 t (hrs)
100 μM
Chk2
RuRuPhen (100 μM)
Cisplatin (40 μM)
Bl Bl 1 3 6 12 24 48 72 L 1 3 6 12 24 t (hrs)
100 μM
β-actin (for p-Chk2)

Figure S9 continued:

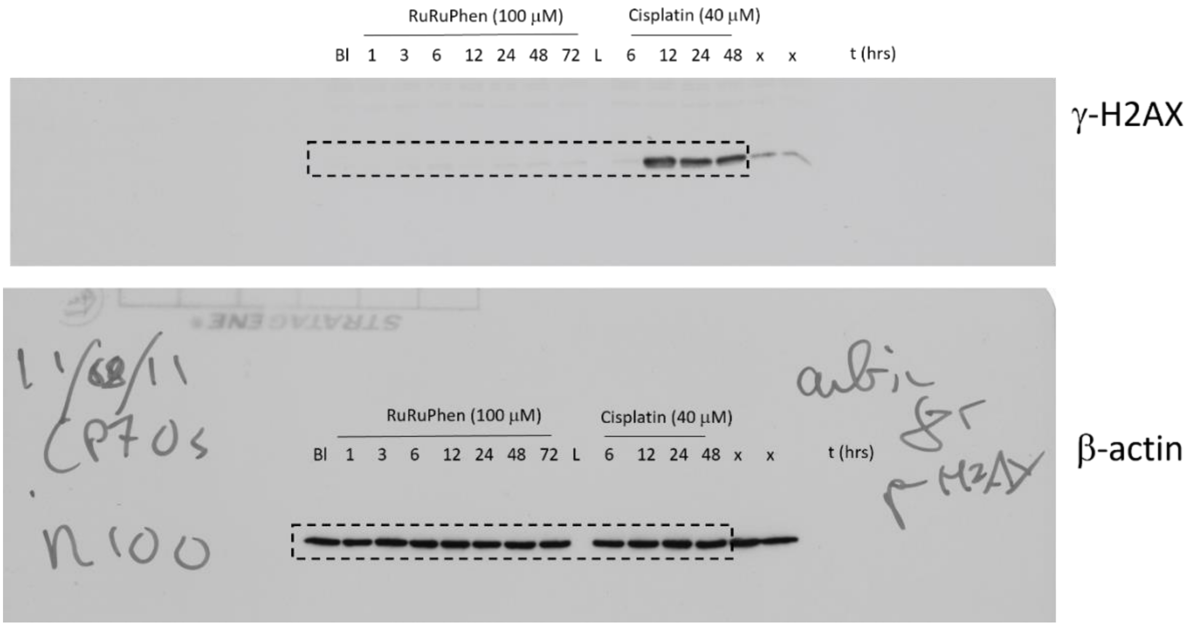

Appendix 2: NMR and MS data.

^{1}H NMR spectrum of RuRuPhen

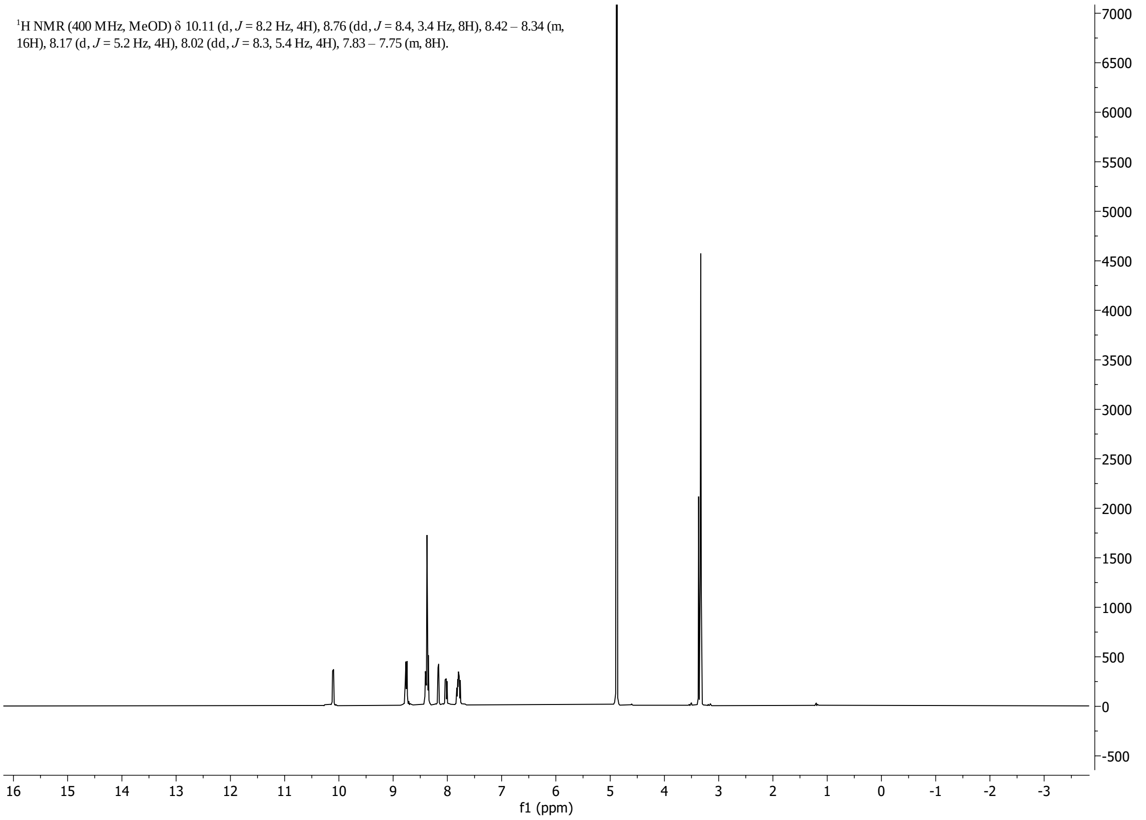

DEPTQ ^{13}C NMR spectrum of RuRuPhen

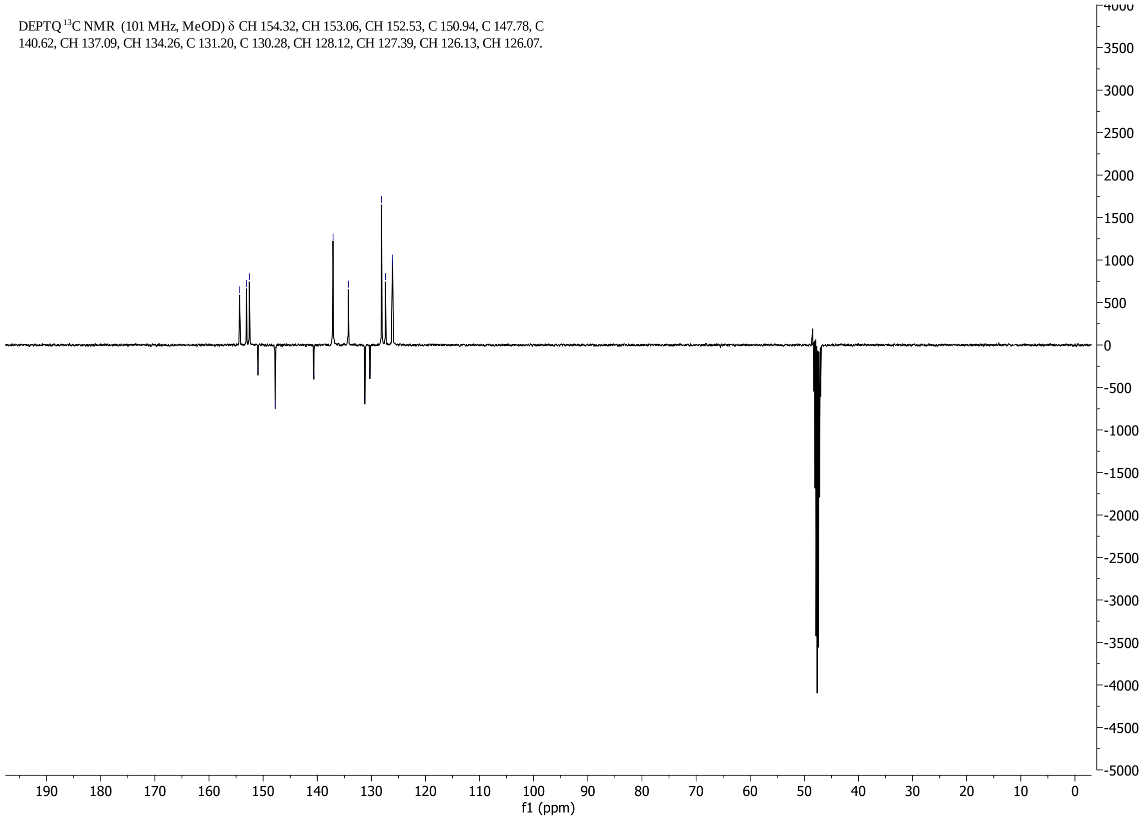

ESI-MS of RuRuPhen

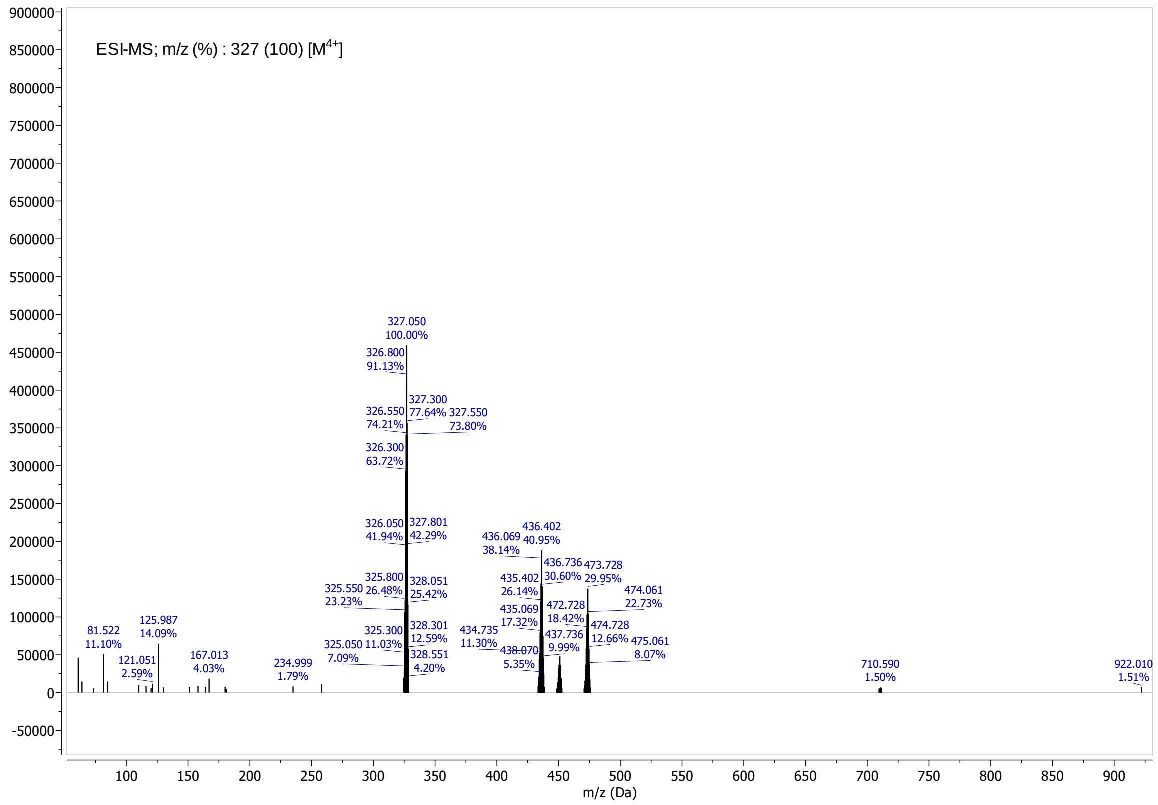

Supplement: Supplementary file 1 — Supporting Information [file ANIE-61-0-s001.pdf]
